# *Viscum album* Exerts Anti-Inflammatory Effect by Selectively Inhibiting Cytokine-Induced Expression of Cyclooxygenase-2

**DOI:** 10.1371/journal.pone.0026312

**Published:** 2011-10-18

**Authors:** Pushpa Hegde, Mohan S. Maddur, Alain Friboulet, Jagadeesh Bayry, Srini V. Kaveri

**Affiliations:** 1 Institut National de la Santé et de la Recherche Médicale, Unité 872, Paris, France; 2 Université de Technologie de Compiègne, Compiègne, France; 3 Centre de Recherche des Cordeliers, Equipe 16- Immunopathology and Therapeutic Immunointervention, Université Pierre et Marie Curie – Paris 6, UMR S 872, Paris, France; 4 Université Paris Descartes, UMR S 872, Paris, France; 5 Université de Technologie de Compiègne, UMR CNRS 6022, Compiègne, France; Centre de Recherche Public de la Santé (CRP-Santé), Luxembourg

## Abstract

*Viscum album* (VA) preparations are extensively used as complementary therapy in cancer and are shown to exert anti-tumor activities which involve the cytotoxic properties, induction of apoptosis, inhibition of angiogenesis and several other immunomodulatory mechanisms. In addition to their application in cancer therapy, VA preparations have also been successfully utilized in the treatment of several inflammatory pathologies. Owing to the intricate association of inflammation and cancer and in view of the fact that several anti-tumor phytotherapeutics also exert a potent anti-inflammatory effect, we hypothesized that VA exerts an anti-inflammatory effect that is responsible for its therapeutic benefit. Since, inflammatory cytokine-induced cyclo-oxygenase-2 (COX-2) and prostaglandin E2 (PGE2) play a critical role in the pathogenesis of inflammatory diseases, we investigated the anti-inflammatory effect of VA on regulation of cyclo-oxygenase expression and PGE2 biosynthesis by using human lung adenocarcinoma cells (A549 cells) as a model. A549 cells were stimulated with IL-1β and treated with VA preparation (VA Qu Spez) for 18 hours. PGE2 was analysed in the culture supernatants by enzyme immunoassay. Expression of COX-2 and COX-1 proteins was analyzed by immunoblotting and the expression of COX-2 mRNA was assessed by semi-quantitative RT-PCR. We found that VA Qu Spez inhibit the secretion of IL-1β-induced PGE2 in a dose-dependent manner. Further, we also show that this inhibitory action was associated with a reduced expression of COX-2 without modulating the COX-1 expression. Together these results demonstrate a novel anti-inflammatory mechanism of action of VA preparations wherein VA exerts an anti-inflammatory effect by inhibiting cytokine-induced PGE2 via selective inhibition of COX-2.

## Introduction

VA preparations are standardized aqueous extracts of *Viscum album L.* (commonly known as European mistletoe, a semi-parasite that grows on different host trees), composed mainly of mistletoe lectins and viscotoxins [1]–[3] and several other biologically active molecules like flavonoids, several enzymes, peptides (such as viscumamide), amino acids, thiols, amines, polysaccharides, cyclitoles, lipids, phytosterols, triterpines, phenylpropanes and minerals [4]. Based on the host tree and the method of extraction, currently at least three VA preparations are available for therapeutic application: VA Qu Spez (oak tree), VA P (pini) and VA M (mali). For a long time, VA preparations have been used as complementary therapy for several types of cancer, mainly in Europe and also to some extent in other parts of the world [5], [6]. Therapeutic benefit of VA preparations when utilized along with surgery, chemotherapy or radiotherapy contributes to the overall improvement in the quality of life in cancer patients [7]–[10]. In addition, VA preparations are also implicated as conventional phytotherapeutics in the treatment of several conditions associated with the nervous system abnormalities, allergic reactions, and immuno-inflammatory disorders [11]–[16].

Despite their use for the past several decades, the precise mechanisms associated with anti-tumoral effects of VA are not completely understood. Several mutually non-exclusive mechanisms have been proposed, which include induction of apoptosis and cytotoxicity in tumor cells and inhibition of process of angiogenesis [10]–[22]. VA preparations also exert several immuno-stimulatory mechanisms by interacting with the cellular and humoral compartments of the immune system thus potentiating an anti-tumor immune response [22]–[30]. However, the mechanisms of action of *Viscum album* underlying their therapeutic benefits in inflammatory pathologies are yet to be explored. In this study, we therefore investigated the anti-inflammatory mechanisms of VA preparations.

Inflammation is a basal physiological phenomenon that occurs as a complex set of responses to an infectious agent or to a tissue injury so as to eliminate the causative agent and to initiate the healing process. It is a physiopathological symptom in a variety of conditions of infectious, autoimmune or of tumoral origin. Interaction of innate and adaptive immune cells as well as non-immune cells such as endothelial cells and fibroblasts with inflammatory stimulus induces the production of several molecular mediators such as cytokines, chemokines, bioactive amines, eicosanoids, and products of proteolytic cascades, such as bradykinin [31]–[37]. These inflammatory mediators acts on various target tissues and exert changes in their homeostatic functions and therefore inflammation has to be kept in check which can be acieved by various anti-inflammatory agents including steroids, non-steroidal antiinflammatory agents, intravenousimmunoglobulins, immunosuppressor cells, neutralizing monoclonal antibodies to inflammatory cytokines [38]–[45].

PGE2, a derivative of eicosanoids, plays an important role as molecular mediator of inflammatory response. Overproduction of PGE2 is observed in many of the human pathologies associated with inflammation and pro-tumoral condition [46]–[49]. PGE2 is synthesized by cyclo-oxygenases, COX-1 (expressed constitutively) and COX-2 (induced in response to inflammatory stimulus). COX-2 is transcriptionaly activated and over expressed in response to many pro-inflammatory stimuli like IL-1β, IFN-γ, TNF-α and also pathogen stimuli such as lipopolysaccharides leading to an enhanced biosynthesis of PGE2 [50]. Therefore, pharmacological strategies to suppress the COX-2 expression and PGE2 secretion are of great interest and are being exploited to develop potent therapeutics to resolve inflammation and several pro-tumor conditions [51]–[56]. Indeed, several phytotherapeutics and plant-derived molecules like curcumin, some of the flavonoids and polyphenols that have anti-tumor properties also exert anti-inflammatory activity by down-regulating the expression of COX-2 and PGE2 biosynthesis [57]–[67]. Though there are few reports showing anti-inflammatory activity of certain molecules isolated from *Viscum album*, precise mechanisms of action associated with its therapeutic benefit are yet to be explored [68], [69].

Here, we therefore examined the hypothesis that VA exerts anti-inflammatory effect by interfering with the expression and biochemical functioning of COX-2 which explains in part, the mechanisms of actions of VA in several inflammatory pathologies.

## Results

### *Viscum album* inhibits cytokine-induced PGE2 secretion in A549 cells

**Figure 1:**
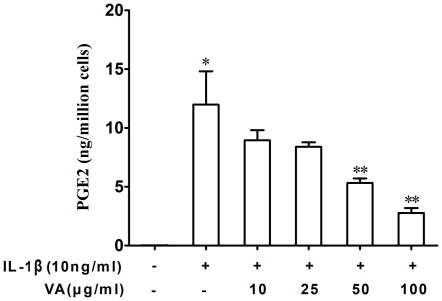
Inhibition of PGE2 secretion by *Viscum album* in A549 cells. Cells were treated with IL-1β (10 ng/ml) and increasing concentrations of VA Qu Spez for 18 hours. PGE2 was analyzed in culture supernatants by EIA. Results are mean±SEM from 4 independent experiments (*p<0.05 versus control cells, **p<0.05 versus cells treated with IL-1β, analyzed by paired Student-t-test).

Human lung adenocarcinoma-derived A549 cells were stimulated with IL-1β for 18 hours in the presence or absence of VA Qu Spez, a therapeutic preparation of *Viscum album* that grows on oak trees. PGE2 was estimated in the culture supernatants by enzyme immunoassay as explained in Materials and Methods. Inflammatory cytokine IL-1β significantly induced the secretion of PGE2. We found that VA Qu Spez inhibits the IL-1β-induced PGE2 secretion in a dose-dependent manner (Figure 1). At 50 µg/ml and 100 µg/ml, VA Qu Spez significantly inhibited the secretion of IL-1β-induced PGE2 secretion.

### *Viscum album* does not inhibit the cytokine-induced COX-2 mRNA expression

**Figure 2:**
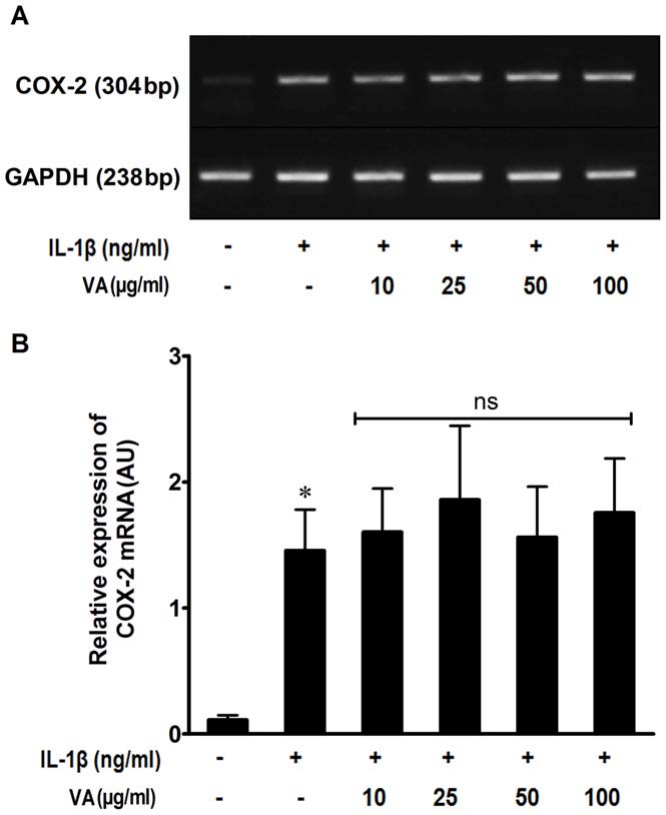
Effect of VA Q Spez on IL-1β-induced COX-2 transcription. Total cellular RNA was isolated from A549 cells that were cultured either in medium alone or stimulated with IL-1β (10 ng/ml) with or without various concentrations of VA for 18 hours. mRNA expression of COX-2 was analyzed by semi quantitative RT-PCR using COX-2-specific primers. Amplification of a house keeping gene GAPDH is used as an internal control. (A) PCR products were separated on 1% agarose gel and a representative gel picture is shown. (B) Relative expression (mean ± SEM) of COX-2 mRNA from six independent experiments as quantified by densitometry (ratio of density of COX-2: that of GAPDH). * P<0.05 analyzed by paired Student-t-test, ns refers to non-significant, compared to IL-1β stimulated cells.

Since the induction of PGE2 by pro-inflammatory cytokines is known to be associated with the over expression of *cox-2* gene, we analyzed the modulation of mRNA expression of COX-2 by VA. IL-1β stimulated-A549 cells were harvested following the treatment with VA Qu Spez and their total cellular RNA was subjected to reverse transcription and polymerase chain reaction. Unstimulated cells showed a basal amount of COX-2 mRNA and it was significantly up-regulated by IL- 1β. However, we found that VA Qu Spez did not inhibit the cytokine-induced COX-2 mRNA expression (Figure 2). Indeed, no significant change in the amount of COX-2 mRNA expression was observed even at the highest concentration of VA (100 µg/ml) used in our experiment.

### *Viscum album* inhibits cytokine-induced COX-2 protein expression

In order to investigate whether VA-mediated inhibition of the cytokine-induced PGE2 secretion (Figure 1) correlated with the reduced level of COX-2 protein, we analyzed the expression of COX-2 protein in IL-1β-stimulated cells following VA treatment, by immunoblotting.

**Figure 3:**
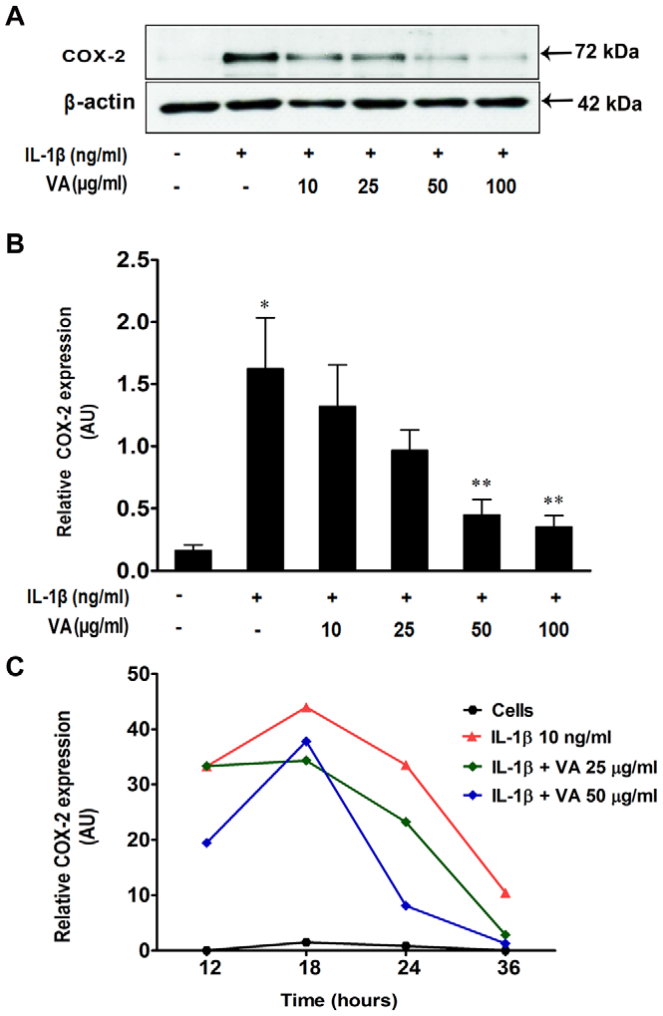
Inhibition of cytokine-induced COX-2 protein expression by *Viscum album* in A549 cells. A549 cells were either cultured in medium alone or treated with IL-1β (10 ng/ml) with or without increasing concentration of VA Qu Spez for 18 hours. Using 20 µg of total cellular protein, expression of COX-2 was analyzed by western blotting. **A.** A representative western-blot depicting the effect of VA on expression of IL-1β-induced COX-2. **B.** Relative expression (mean ± SEM) of COX-2 protein from four independent experiments as quantified by densitometry (ratio of density of COX-2: that of β-actin). *P<0.05 versus control cells and ** *p*<0.05 versus IL-1β-stimulated cells as analyzed by paired Student-t-test. **C.** Kinetics of COX-2 protein expression upon treatment of A549 cells with various doses of VA. The cells were either cultured in medium alone or stimulated with IL-1β with our without VA for 12, 18, 24 and 36 hours. Expression of COX-2 protein was analyzed by western blotting and relative expression of COX-2 protein was quantified by densitometry (ratio of density of COX-2: that of β-actin). Results are representative of two experiments.

Figure 3A depicts a representative blot of the expression pattern of COX-2 in different culture conditions. Protein expression was quantified by using densitometry analysis of the blots from 4 independent experiments and relative COX-2 expression normalized to that of β-actin is shown in the histogram (Figure 3B). IL-1β significantly induced the expression of COX-2 protein. Of interest and in contrast to mRNA profiles, VA Qu Spez inhibited the IL-1β-induced expression of COX-2 protein in a dose-dependent manner. Furthermore, we followed the expression profiles of IL-1β induced COX-2 in the presence and absence of VA at different time points (12, 18, 24 and 36 hours) following cytokine stimulation. Kinetic analysis of COX-2 at two different concentrations of VA is shown in Figure 3C. IL-1β induced the maximum expression of COX-2 at 18 hours and then onwards, the expression was gradually declined. At 25 µg /ml, VA inhibited the COX-2 protein expression at 18, 24 and 36 hours and at 50 µg/ml, inhibited COX-2 expression at 18 hours and more drastically after 24 hours.

### *Viscum album* does not modulate the expression of COX-1 protein

**Figure 4:**
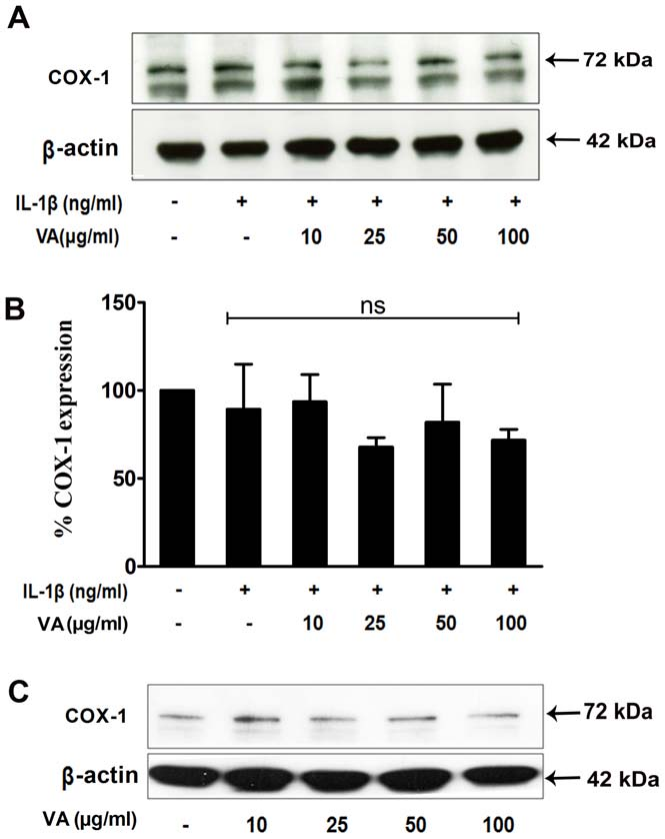
Effect of *Viscum album* on COX-1 expression in A549 cells. **A and B.** A549 cells were treated with increasing concentrations of VA Qu Spez in presence of IL-1β (10 ng/ml) for 18 hours. Protein expression of COX-1 was analyzed by western blot. A representative western-blot depicting the effect of VA on COX-1 expression under IL-1β-stimulated culture conditions (A). Relative expression (mean ± SEM) of COX-1 protein from three independent experiments as quantified by densitometry (ratio of density of COX-1: that of β-actin). ns, non-significant (B). **C.** Cells were cultured either in medium alone or treated with increasing concentrations of VA Qu Spez without IL-1β for 18 hours. 20 µg of total cellular protein was separated by 10% SDS-PAGE followed by western blotting to analyze the expression of COX-1. Results are representative of two experiments.

**Figure 5:**
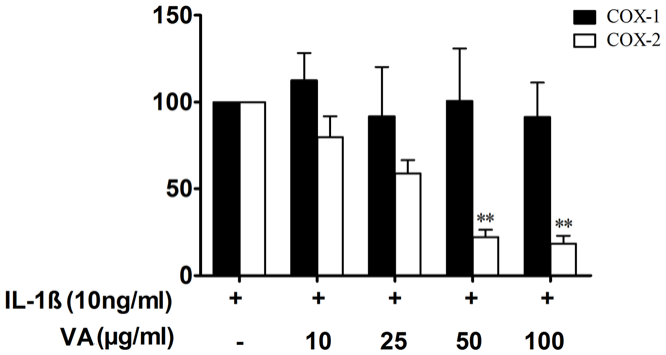
Selective inhibition of COX-2 expression by *Viscum album*. Quantitative comparison between expression of COX-1 and COX-2 proteins as revealed by western blot. Protein expression was quantified by densitometry and expressed as percentage protein expression normalized to that in cells stimulated with IL-1β. Results (mean ± SEM) obtained from 3 independent experiments are presented. * *P*<0.05 versus control cells for COX-2 and ** *p*<0.05 versus IL-1β-stimulated cells for COX-2 as analyzed by paired Student-t-test. The values for COX-1 are non-significant.

Since both COX-1 and COX-2 play an important regulatory role in the biosynthesis of PGE2, we then analysed the effect of VA on the expression of COX-1 protein, which is a constitutively expressed isoform of cyclo-oxygenase and is implicated in the functional homeostasis of the cell. We found that A549 cells constitutively express COX-1 and the expression of which was not modified upon treatment of cells with IL-1β (Figure 4A). However, VA Qu Spez did not modulate either the constitutive expression of COX-1 protein nor the expression of COX-1 in IL-1β-stimulated cells (Figure 4A and B). As VA displayed a strong inhibitory effect on IL-1β-induced COX-2 expression without modulating the COX-1 level, our results demonstrate that VA selectively inhibits the expression of COX-2 (Figure 5).

## Discussion

The therapeutic benefit of VA in diverse pathologies is attributed to the method of preparation, the proportion of various bioactive compounds present within the extracts and also to the host trees. Several lines of evidence have revealed that VA preparations exert an anti-tumoral effect because of their ability to induce apoptosis and to inhibit cell proliferation, providing a strong basis for their application as complementary therapy in cancer. However, successful utilization of these preparations in the treatment of certain inflammatory pathologies raises several questions related to their mechanisms of action. With the aim of understanding the role of VA in modulating the immuno-inflammatory response, we analysed the effect of VA on the PGE2 axis and its regulation at cyclo-oxygenase level.

Cyclo-oxygenases are the regulatory enzymes of PGE2 biosynthetic pathway which catalyse the rate limiting step. They convert the free arachidonic acid in the cell cytosol obtained upon degradation of membrane phospholipids into prostaglandin H2 (PGH2), an active precursor for the synthesis of various prostanoids. Among the isoforms of cyclo-oxygenases, COX-1 is constitutively expressed in the cells whereas COX-2 is induced in response to inflammatory stimuli and contributes majorly to the induction of PGE2 [70]. PGE2 is a molecular mediator of several homeostatic functions including those of gastric mucosa and vascular endothelium [71]. However, it also exerts potent pro-inflammatory effects including the induction fever and pain. Overproduction of PGE2 occurs in response to pro-inflammatory stimuli and correlates with the severity of certain infectious and inflammatory conditions [46]–[49]. PGE2 exerts autocrine and paracrine actions on the target cells and can induce pro-inflammatory reactions. Cyclo-oxygenases have thus constituted attractive targets for designing the anti-inflammatory molecules such as non-steroid anti-inflammatory drugs (NSAID) and for the conception of novel phytotherapeutics [51], [52].

Our results demonstrate that the anti-inflammatory function of VA implicates inhibition of IL-1β-induced PGE2 biosynthesis. In order to understand the mechanisms underlying the VA-mediated inhibition of PGE2 secretion, we analyzed the expression pattern of cytokine-induced COX-2 in the presence of VA. RT-PCR results confirmed that IL-1β induces the expression of COX-2 mRNA while VA does not modulate this cytokine-induced expression of COX-2 mRNA. On the contrary, VA significantly inhibited COX-2 protein expression induced by IL-1β. These results suggest that VA exerts a post-transcriptional regulatory effect on COX-2 expression [72].

The effect of VA was not restricted to IL-1β-induced COX-2 alone. VA also showed an inhibitory effect on IFN-γ and TNF-α-induced COX-2 expression (data not shown). Since, inflammation is a consequence of signaling of various inflammatory cytokines released by innate and adaptive immune cells, endothelial cells and epithelial cells, our results thus indicate that suppression of COX-2 by VA is not restricted to an inflammation created by a particular cytokine rather VA inhibits COX-2 expression mediated by a wide range of cytokines. This inhibitory effect of VA at the protein expression level suggests its probable action in interfering with the process of translation resulting in the quantitative reduction of the protein. Further, kinetic analysis of COX-2 expression induced by IL-1β showed a typical time-dependant increase in protein expression reaching a maximum at 18 hours followed by a decline at later time points. At each time point, treatment of the cells with VA resulted in the reduced expression of COX-2 induced by IL-1β. This reduction in the presence of VA was more pronounced at 24 and 36 hours as compared to cells treated with IL-1β alone indicating a possible effect of VA on protein degradation. Together, modulation of COX-2 by VA strongly favours the role of VA as an anti-inflammatory therapeutic.

From the viewpoint of an anti-inflammatory therapeutic, inhibition of PGE2 synthesis by acting at the level of cyclo-oxygenases exposes a major concern of reducing the homeostatic levels of PGE2 because of the simultaneous inhibition of COX-1 and COX-2. Owing to the structural and functional homology between the COX-1 and COX-2, most of the NSAID which are synthetically designed to inhibit the enzymatic activity of COX-2 also inhibit COX-1, thereby affecting the overall level of PGE2 and are therefore known to cause several severe side effects [73]. Thus, specific inhibitors of COX-2 represent attractive therapeutic alternatives for several inflammatory pathologies [51]. Interestingly, in our study, at all the concentrations of VA which inhibited COX-2, we did not observe any change in the expression of COX-1, irrespective of a robust stimulation by IL-1β. These findings suggest a strong selectivity in anti-inflammatory mechanism of VA by inhibiting COX-2 expression.

Long-term side effects of NSAID in various pathological conditions and the increasing body of evidence for anti-inflammatory activity of plant-derived molecules together encourage the conception of phytotherapeutics as potent alternatives to classical anti-inflammatory drugs [73], [74]. Several clinical studies have revealed the selectivity of certain plant-derived molecules in inhibiting COX-2 that are as efficient as synthetic COX-2-specific antagonists (rofecoxib and celecoxib) in rescuing from both acute and chronic inflammatory conditions and some of these plant-derivatives are superior to synthetic molecules in their anti-inflammatory and analgesic effect [74], [75]. With the growing interest of promising new generation anti-inflammatory therapeutics, exploring and characterizing the novel phytotherapeutics with strong selectivity towards COX-2 are of great value. Together, our results demonstrate that VA exerts an anti-inflammatory effect by interfering with the cytokine-induced PGE2 biosynthesis through a selective inhibition of COX-2 protein suggesting its beneficial role with minimal side effects. These results are relevant in understanding the mechanism of action of VA and may provide an insight for further exploration of anti-inflammatory mechanisms of VA and other plant-derived molecules in diverse pathologies.

## Materials and Methods

### *Viscum album* preparations

VA Qu Spez was a kind gift from Weleda AG (Arlesheim, Switzerland). VA Qu Spez is a therapeutic preparation of *Viscum album* growing on oak trees and is obtained as an isotonic solution of 5 mg/ml and 10 mg/ml formulated in 0.9% NaCl. It is free from endotoxin and contains the standardized levels of mistletoe lectins (0.375 ng/ml) and viscotoxins (0.12 ng/ml).

### Culture of A549 cells

Human lung adenocarcinoma cell line A549 was a kind gift from Dr. Maria Castedo-Delrieu, Institute Gustave Roussy, Villejuif, France. A549 cells were grown in 75 cm^2^ culture flasks in Dulbecco's modified Eagle's medium (DMEM) F-12, GIBCO®, BRL Life Technologies, Grand Island, NY, USA)) supplemented with 10% fetal calf serum and 50 U/ml penicillin and 50 µg/ml of streptomycin (GIBCO®, BRL, Cergy Pontoise, France). Cells are incubated at 37°C with 5% CO_2_ in humidified atmosphere to obtain the cells of about 80–90% confluence and used for all experiments.

### Induction of COX-2 and treatment with VA Qu Spez

Cells grown in complete medium were harvested by tripsinisation using 0.5% trypsin (Biological Industries, Kibbutz Beit Haemek, Israel) and are seeded in six well culture plates (1×10^6^ cells per well), and incubated at 37°C till they reach confluence of 80–90%. Wells containing the adherent A549 are then replenished with the complete medium containing recombinant human IL-1β (10 ng/ml) (Immuno Tools, Friesoythe, Germany) in the presence and absence of VA Qu Spez and incubated for 18 hours at 37°C and 5% CO_2_. After 18 hours of incubation culture supernatants were collected and used for the estimation of PGE2 and cells are used for estimating the mRNA and protein levels of COX-2/COX-1.

### Enzyme immunoassay for PGE2

PGE2 is estimated in the culture supernatants by using competitive enzyme immunoassay (EIA) kit (Cayman Chemicals Co, Ann Arbor, MI, USA) according to the manufacturer's instructions. Cell free supernatants were analysed for the free PGE2 released.

### Isolation of total RNA and RT-PCR

Total cellular RNA was isolated using RNA isolation kit (Invitrogen, Life technologies, France) according to the manufacturers' instructions. 0.2 µg of total RNA was reverse transcribed to obtain the cDNA using the Superscript II from (Invitrogen, Life technologies, France) Semi-quantitative PCR is carried out using COX-2 specific primers. Amplification of housekeeping gene GAPDH is used as an internal control. Oligonucleotide primers were purchased from Eurogentech, France. Sequences of oligonucleotides are as given in Table 1.

**Table 1:** Oligonucleotide primers used in the study.

Human COX-2 (sense) 5′-TTCAAATGAGATTGTGGGAAAATTGCT-3′
Human COX-2 (antisense) 5′-AGATCATCTCTGCCTGAGTATCTT-3′
Human GAPDH (sense) 5′GAGTCAACGGATTTGGTCGT3′
Human GAPDH (antisense) 5′TTGATTTTGGAGGGATCTCG-3′

### Immunoblotting of COX-2 and COX-1

Following the appropriate treatment, cells were harvested by a mild tripsinisation and washed with 1× phosphate buffered saline. Cells were lysed using lysis buffer containing 50 mM Tris-HCl (pH 7.4), 0.25% sodium deoxycholate, 150 mM NaCl, 1 mM EDTA, 1% NP-40, 1 mM PMSF and 1× protease inhibitor cock-tail (Sigma-Aldrich, Lyon, France). Cells were suspended in the lysis buffer (100 µl/million cells) and incubated on ice for 30 mins. Supernatants were collected following the centrifugation at 13200 rpm for 20 mins. Total cellular protein is estimated by Bradford method and 20 µg of each sample is loaded on SDS polyacrylamide gel and subjected for electrophoretic migration. Proteins separated on gel were then transferred on to an activated PVDF membrane by semi-dry western transfer (2 hours at 50 mA). Non-specific binding of antibodies is blocked by treating the membrane with 5% non fat milk in tris buffered saline with tween 20 (TBST-20 mM Tris- HCl (pH 7.4), 137 mM NaCl and 0.1% Tween 20) for 2 hours at room temperature. Membranes were incubated with primary antibodies diluted according to the manufactures' instructions in 5% BSA overnight at 4°C. Following the three washes with TBST, Blots were then treated with HRP-conjugated secondary antibodies in 5% BSA (1/4000) for 2 hours at room temperature. Primary antibodies against human COX-2, β-Actin, and the HRP labelled secondary antibodies were procured from Cell Signalling Technology (Ozyme, France). Human anti-COX-1 is obtained from Santa Cruz Biotechnology (CA, USA). Blots were washed well with TBST with minimum three changes for an hour and then revealed using ECL plus western lightening reagent (Perkin Elmer, Waltham, MA, USA) according to manufactures' instructions.

### Statistical analysis

Densitometric analysis of the immunoblots was performed using BIO-1D analysis software. Densitometric values were expressed as arbitrary units and subjected for statistical analysis by paired Student-t- test. All the observations are expressed as Mean±SEM. Graphpad Prism 5.0 is used for all the statistical analysis. *P* values less than 0.05 were considered to be statistically significant.
